# AI-Assisted Impedance Biosensing of Yeast Cell Concentration

**DOI:** 10.3390/bios16010018

**Published:** 2025-12-25

**Authors:** Amir A. AlMarzooqi, Mahmoud Al Ahmad, Jisha Chalissery, Ahmed H. Hassan

**Affiliations:** 1 Department of Biology, College of Sciences, United Arab Emirates University, Al Ain 15551, United Arab Emirates; 201914725@uaeu.ac.ae; 2 Department of Electrical and Communication Engineering, United Arab Emirates University, Al Ain 15551, United Arab Emirates; 3 Department of Biochemistry and Molecular Biology, College of Medicine and Health Sciences, United Arab Emirates University, Al Ain 15551, United Arab Emirates; jishachalissery@uaeu.ac.ae (J.C.); ahmedh@uaeu.ac.ae (A.H.H.)

**Keywords:** impedance spectroscopy, optical density, machine learning, yeast

## Abstract

Quantifying microbial growth with high temporal resolution remains essential yet challenging due to limitations of optical, manual, and biochemical methods. Here, we introduce an AI-enhanced electrochemical impedance spectroscopy platform for real-time, label-free monitoring of *Saccharomyces cerevisiae* growth. Broadband impedance measurements (1 Hz–100 kHz) were collected from yeast cultures across log-phase development. Engineered features—derived from impedance magnitude and phase—captured dielectric and conductive shifts associated with cell proliferation, membrane polarization, and ionic redistribution. A Gaussian Process Regression model trained on these features predicted optical density (OD600) with high precision (RMSE = 0.79 min; R^2^ = 0.9996; r = 0.9998), and achieved 100% classification accuracy when discretized into 15-min growth intervals. The system operated with sub-millisecond latency and minimal memory footprint, enabling embedded deployment. Benchmarking against conventional methods revealed superior throughput, automation potential, and independence from labeling or turbidity-based optics. This AI-driven platform forms the core of a real-time digital twin for yeast culture monitoring, capable of predictive tracking and adaptive control. By fusing electrochemical biosensing with machine learning, our method offers a scalable and robust solution for intelligent fermentation and bioprocess optimization.

## 1. Introduction

Monitoring yeast cell growth and physiology is vital for biotechnological processes, requiring efficient process management and continuous control of cellular development [1]. Optical density (OD), commonly measured at 600 nm (OD_600_), serves as the standard for estimating cell concentration. However, conventional OD methods face challenges, including light scattering, turbidity, and an inability to distinguish between live and dead cells, making accurate assessment of yeast populations difficult [2]. Electrochemical impedance analysis (EIA) is emerging as a promising alternative to traditional optical methods by analyzing cell suspension electrochemical characteristics across a broad frequency range [3]. EIA provides key insights into cellular properties, including membrane integrity, intracellular composition, and morphology [4,5]. This technique strongly correlates with yeast cell concentration and viability, offering a reliable, non-invasive method for real-time monitoring of cell growth [6].

In parallel with advances in sensing technologies, artificial intelligence (AI) has become an indispensable tool across modern chemistry and biotechnology. Machine learning methods have been widely applied to analyze complex spectroscopic and analytical datasets including Raman spectra, NMR signals, mass spectrometry profiles, and electrochemical measurements, often outperforming traditional chemometric approaches in accuracy and robustness [7,8,9]. AI-driven models have further enabled enhanced chemical quantification, improved noise suppression, and higher sensitivity in analytical measurements [10,11]. Within bioprocessing, machine learning is increasingly used for real-time prediction of biomass, viability, metabolic state, and fermentation performance, supporting automated control and optimization of cell culture systems [12,13,14,15]. These developments highlight the transformative role of AI in interpreting high-dimensional chemical and biological data and underscore the need for data-driven frameworks that can complement or replace conventional optical measurements.

Building upon previous work involving the electrochemical characterization of yeast cells via impedance spectroscopy and circuit-based modeling approaches [16,17], nonlinear electrochemical responses in yeast suspensions have been shown to intensify with increasing voltage amplitude and decreasing signal frequency [18]. These findings also supported the feasibility of applying electrical treatments—specifically irreversible electroporation—as non-thermal strategies for yeast inactivation in food systems without compromising quality attributes [19,20].

Impedance-based measurements were shown to be reliable in capturing yeast cell dynamics, offering a complementary or alternative pathway to traditional optical techniques [21]. Additional investigations incorporated microfluidic and optoelectronic configurations—such as Fabry–Perot cavity-based sensing [22] and spatially resolved impedance mapping at designated frequencies [23]—to track physiological and spatial changes in yeast populations. Earlier electrochemical monitoring tools primarily focused on endpoints like metabolic activity [24]; however, ongoing research seeks to expand their capability by integrating multi-modal sensing frameworks.

A hybrid real-time monitoring system has also been proposed, combining optical and impedance modalities to enable more comprehensive physiological profiling. In this context, machine learning was identified as a valuable asset for extracting meaningful patterns from high-dimensional data, while lumped-element circuit modeling proved effective in simulating electro treatment effects and benchmarking electrical outputs against optical references. Moreover, capacitance–voltage profiling demonstrated the potential to monitor yeast cell cycle progression and estimate doubling times through dielectric signature analysis [25].

The EIA technique strongly correlates with yeast cell concentration and viability, offering a reliable, non-invasive method for real-time monitoring of cell growth [26]. Advancements in machine learning have further enhanced the potential of EIA for microbial monitoring [27]. Machine learning algorithms, particularly neural networks, can analyze complex patterns in impedance data, enabling highly accurate predictions of OD_600_ values without direct optical measurements [28]. Furthermore, the use of machine learning models enhances sensitivity, enabling the early detection of changes in yeast cell behavior, and providing a scalable framework for industrial applications [29].

Electrochemical impedance provides rich, multi-frequency information about yeast growth. However, the relationship between impedance signatures and cell concentration is nonlinear and sensitive to multiple experimental factors [30]. As emphasized, bioimpedance is inherently a multivariable measurement. This makes AI well suited to extract meaningful patterns and improve the prediction of biological states from complex datasets [31]. By combining AI with impedance measurements, it becomes possible to achieve accurate prediction from complex impedance signatures. At the same time, AI can compensate for experimental variability and automatically detect and correct artifacts [32]. This integration strengthens robustness in real-time or high-throughput applications and enhances the interpretability of bioimpedance-based predictions [33]. Ultimately, merging AI with electrochemical impedance transforms yeast biosensors from simple measurement tools into intelligent analytical systems capable of delivering reliable and high-resolution biological monitoring.

This study explores the integration of EIA with machine learning to estimate cell density of yeast cell suspensions, facilitating real-time monitoring of growth. EIA can effectively capture changes in cell density during yeast growth by measuring variations in electrochemical impedance that result from increasing cell numbers. As yeast cells accumulate, they alter the electrical conductivity and permittivity of the suspension, particularly at low frequencies, enabling real-time, non-invasive monitoring of culture density. The incorporation of machine learning enhances the efficiency and accuracy of cell density measurement, providing deeper insights into yeast growth and streamlining cell culture monitoring.

## 2. Materials and Methods

A. Yeast Sample Preparation

The BY4741 strain (Euroscarf, Oberursel/Germany) of budding yeast was cultured overnight in 5 mL of YPD medium (1% yeast extract, 2% peptone, and 2% dextrose) at 30 °C with orbital shaking at 200 RPM. The following morning, it was subcultured in fresh YPD media and was grown to log phase at 30 °C. The OD600 of the yeast culture was then measured every 20 min using a NanoDrop 2000c spectrophotometer (Thermo Scientific, MA, USA) [34]. Cultures with high cell density were appropriately diluted prior to OD measurement, and the original optical density was subsequently back-calculated based on the dilution factor.

B. Electrochemical Measurements

The experiments utilized a cuvette with dimensions of 10 mm × 21 mm × 4 mm, equipped with electroporation electrodes. Yeast cells were cultured, and the cuvette was filled either with the media or yeast suspension (cells + media) for analysis. For electrochemical measurements, a two-electrode setup was employed to analyze a yeast suspension treated as a dielectric material. The measurements were performed every 20 min using a Gamry Reference 3000 analyzer (Gamry Instruments, PA, USA), capturing impedance magnitude and phase across a frequency range of 1 Hz to 0.1 MHz [35]. The Gamry Reference 3000 determines impedance by applying a sinusoidal excitation and simultaneously recording the voltage and current responses. Using digital phase-sensitive detection, it extracts the amplitudes and phases of both waveforms. The impedance magnitude is calculated as the ratio of the voltage amplitude to the current amplitude. The phase angle is obtained as the difference between the voltage and current phases.

**Figure 1:**
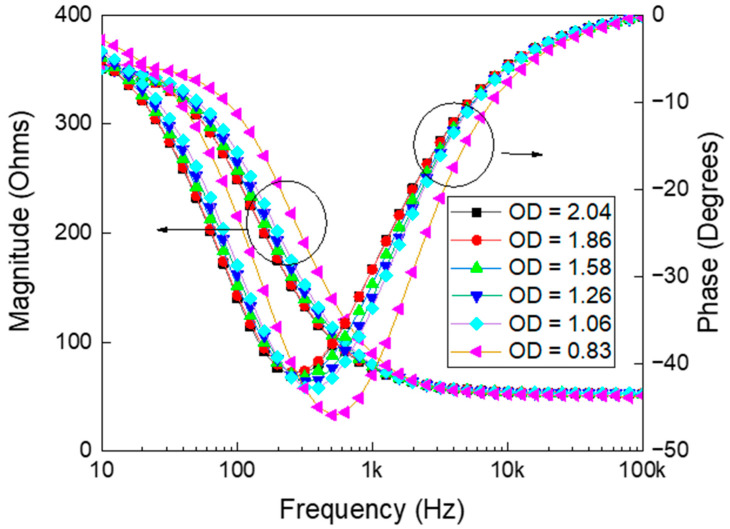
Electrochemical impedance measurements showing both magnitude (left *y*-axis) and phase angle (right *y*-axis) as a function of frequency for samples with varying optical densities (OD). The data illustrate how changes in OD (ranging from 0.83 to 2.04) influence the frequency-dependent electrochemical behavior of the sample. As OD increases, both impedance magnitude and phase response exhibit noticeable shifts, reflecting changes in the underlying material or cellular properties. These trends are critical for characterizing the electrochemical properties of optically dense media.

Simultaneously, the OD_600_ was recorded for each suspension. The relationship between measured electrochemical impedances and the frequency, corresponding to various ODs are depicted in Figure 1. The growth of yeast cells alters both impedance magnitude and phases due to changes in their biological and physical properties, which alter the electrochemical behavior of the suspension [36]. As yeast cells proliferate, their growing size, increasing number, and heightened metabolic activity alter the ionic distribution in the medium, affecting its conductivity and permittivity [37]. These changes, in turn, influence the impedance magnitude and phase, respectively. The cell membrane, acting as a dielectric barrier, contributes to variations in resistive and capacitive properties, which are reflected in the impedance measurements [38].

**Figure 2:**
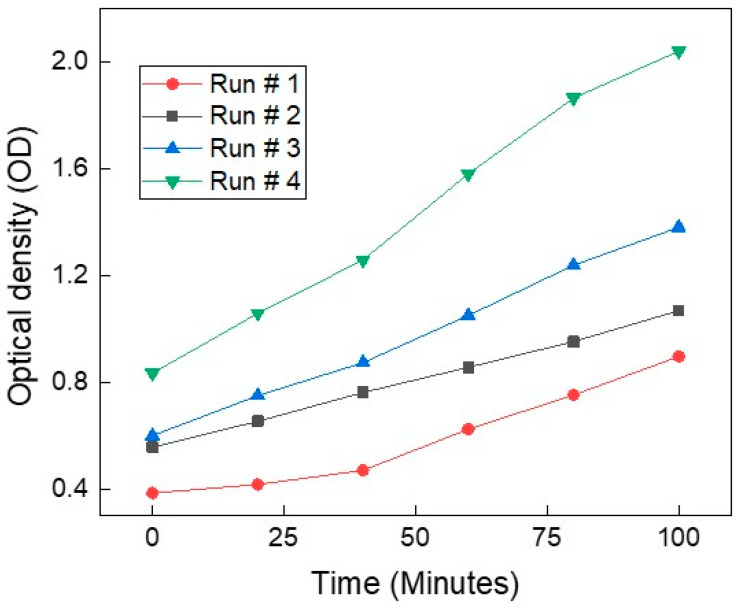
Optical density (OD) measurements over time from four independent yeast growth experiments (Run #1 to Run #4). Despite using the same yeast strain and growth conditions, variability in the growth trajectories is evident across runs. All curves show an upward trend, indicating active proliferation.

Figure 2 presents the optical density trajectories over time from four independent yeast growth experiments. Although all runs were conducted under nominally identical conditions using the same strain, the growth rate differ across experiments. This variation illustrates a common challenge in biological experiments. Such inconsistencies highlight the limitations of purely statistical approaches based on repeat averaging, which may not generalize well. Instead, artificial intelligence models trained on diverse growth trajectories offer a more robust solution. By learning patterns across variable starting points, the AI can predict yeast growth dynamics independent of initial conditions. This makes AI-based systems especially valuable for real-time monitoring and modeling in complex biological settings. An AI-based approach is therefore essential, as it learns invariant relationships between impedance features and growth dynamics across varying initial concentrations, enabling robust and generalizable yeast growth estimation that is insensitive to starting conditions.

**Figure 3:**
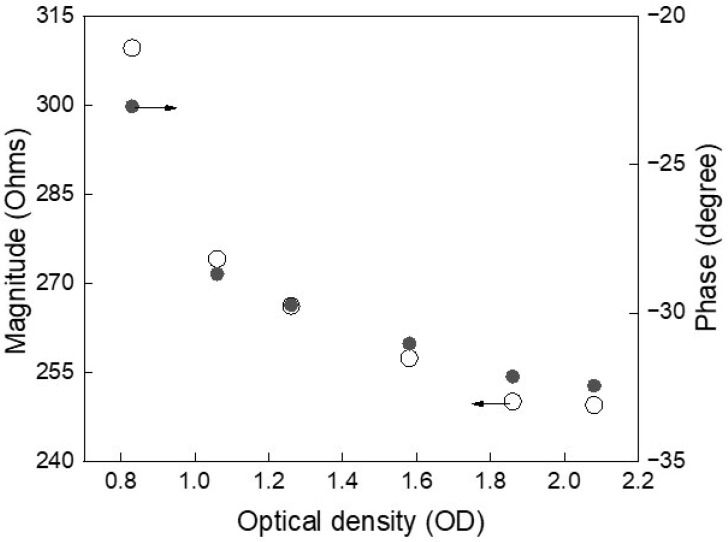
Impedance magnitude decreases and phase shifts negatively with increasing cell concentration, indicating strong correlation with yeast growth and enabling label-free, real-time monitoring.

Figure 3 illustrates the relationship between impedance features and optical density (OD), used here as a proxy for yeast cell concentration. As OD increases—indicating a rise in the number of cells—the impedance magnitude exhibits a consistent decrease, reflecting enhanced ionic conductivity due to cell proliferation. Simultaneously, the phase angle becomes more negative, signaling increased membrane polarization and intracellular complexity. These correlated trends demonstrate the sensitivity of impedance spectroscopy to physiological changes during growth. Despite initial variability among experimental runs, the consistent monotonic behavior of both magnitude and phase reinforces their reliability as non-invasive electrical markers of cell concentration. Once more, these findings support the feasibility of using AI to model such features for continuous, label-free tracking of microbial growth dynamics.

C. AI Methodology

This subsection presents the machine learning framework designed to analyze impedance data for predicting optical density. Input features include impedance magnitudes, phases, and their statistical descriptors, while optical density values serve as the target variable. To translate the electrochemical impedance measurements into accurate estimates of yeast optical density (OD_600_), a structured machine learning (ML) workflow was developed. This workflow was designed to ensure clarity, reproducibility, and interpretability for readers whose primary expertise lies in biosensing rather than artificial intelligence. The complete procedure—spanning data preparation, feature engineering, model training, and evaluation—is described in detail below.

### 2.1. Data Organization and Preparation

Each measurement cycle produced a full impedance spectrum consisting of paired magnitude $\left|Z\right|,$ and phase θ values across the frequency range of 1 Hz to 0.1 MHz. These measurements were synchronized with the corresponding OD_600_ recorded using the spectrophotometer. For machine learning purposes, each frequency point was treated as an individual sample with the following feature vector:

(1) $$\chi =\left[f,\left|Z\right|,\theta \right]$$

and a corresponding target output:

(2) $$y={OD}_{600}$$

This resulted in a high-dimensional dataset in which electrochemical signatures at multiple frequencies were linked directly to yeast cell concentration. Before modeling, the following preprocessing steps were applied: All input features were normalized using z-score scaling, and the impedance measurements were carefully aligned with their corresponding optical density values. Anomalous electrochemical readings were identified and removed through outlier detection procedures. Finally, the dataset was partitioned into an 80% training set and a 20% testing set to ensure unbiased model evaluation.

### 2.2. Feature Engineering and Rationale

Electrochemical impedance measurements carry biologically relevant information linked to membrane capacitance, intracellular conductivity, and the ionic strength of the surrounding medium. To more effectively represent these relationships within the model, several engineered features were introduced. One of the most informative was the combined electrochemical-based descriptor

(3) $$\Psi =\left|Z\right|\times \theta$$

which enhances sensitivity to low-frequency dielectric behavior associated with membrane polarization. Additional frequency-normalized parameters were used to accommodate the logarithmic nature of impedance spectra, and statistical descriptors such as the mean and standard deviation were extracted from defined frequency regions to increase robustness against localized variability. Together, these engineered features capture nonlinear patterns that cannot be resolved through simple linear representations, thereby enhancing the overall predictive performance of the model.

### 2.3. Optical Density Binning for Classification Tasks

To evaluate the model’s capacity to distinguish between different stages of yeast growth, the continuous time values were discretized into uniform intervals of 15 min, covering a total duration of 0 to 120 min. This yielded nine distinct growth stages corresponding to the following intervals: {0, 15, 30, 45, 60, 75, 90, 105, and 120 min}. Each sample in the dataset, derived from impedance statistics (mean and standard deviation of magnitude and phase at each frequency), was labeled with one of these target intervals. This interval-based labeling enables the Gaussian Process Regression (GPR) model [39] to be evaluated not only in terms of continuous time estimation, but also by “rounding” its output to the nearest 15-min bin, mimicking practical classification of biological states in real-time monitoring. Unlike classical curve-fitting methods, the trained Gaussian Process Regression model captures nonlinear variations in yeast growth under different initial conditions, enabling generalized and robust predictions. The interval accuracy thus represents the proportion of test samples correctly classified into their respective time bins, supporting the applicability of the model in automated bioprocess control environments where distinct physiological phases are often mapped to discrete temporal milestones.

### 2.4. Model Selection and Training Procedure

Gaussian Process Regression (GPR) was employed to model the time evolution of yeast growth based on engineered impedance features derived from multi-frequency spectroscopy. The input features included mean and standard deviation values of both impedance magnitude and phase across frequency, as well as composite descriptors such as the product of magnitude and phase (|Z|·θ), capturing coupled electrical characteristics associated with cellular proliferation. Frequencies were log-transformed to reduce skew and enhance sensitivity to biologically relevant transitions. The dataset was constructed from nine time points (0 to 120 min in 15-min steps), and each frequency–time combination formed a separate sample.

The regression model was trained using an automatic relevance determination squared exponential kernel, which allows differential weighting of features and captures smooth, nonlinear dependencies inherent to biological systems. Model performance was evaluated using an 80/20 hold-out strategy, and prediction confidence intervals were obtained from the posterior variance of the GPR model. Key performance metrics included RMSE, MAE, R^2^, and Pearson correlation. In addition to continuous regression accuracy, predictions were also evaluated in discretized time intervals to mimic classification-style labeling often used in bioprocess monitoring. Rounded predictions to the nearest 15-min bin enabled calculation of interval-level accuracy and a confusion matrix across the full growth timeline. This dual-level evaluation confirmed the robustness of the model in capturing both continuous dynamics and discrete phase transitions in yeast culture progression.

### 2.5. Dataset Size and Composition

The dataset was constructed from statistical features extracted at nine measurement intervals spanning 0 to 120 min, in 15-min increments. For each time point, impedance spectra were recorded across 65 logarithmically spaced frequencies ranging from 1 Hz to 0.1 MHz. At each frequency, the mean and standard deviation of the impedance magnitude and phase were computed based on three independent experimental replicates. Additional engineered descriptors, including the product of magnitude and phase (|Z|·θ), were included to enhance sensitivity to growth-related electrical signatures. These features were log-transformed where appropriate to improve linear separability and reduce spectral skew.

Each frequency–time combination constituted an individual sample, yielding a total of 3510 samples (65 frequencies × 9-time intervals × 1 sample per feature set). After excluding incomplete or zero-variance entries, 3510 valid samples remained. The dataset was randomly partitioned into 80% for training and 20% for testing using stratified holdout sampling. Standardization (z-score normalization) was applied to the training set and propagated to the test set to ensure consistent scaling. Internal performance evaluation and hyperparameter optimization were conducted within the training partition, removing the need for a separate validation set. The final composition of the dataset thus offered a robust statistical basis for training and evaluating regression models across a continuous spectrum of growth times, independent of initial optical densities. A representative subset of the impedance dataset is shown in Table 1, spanning measurement intervals from t_0_ to t_8_, which correspond to optical density values ranging from approximately 0.8 to 2.8 OD_600_, capturing key phases of yeast proliferation.

## 3. Results and Discussion

**Figure 4:**
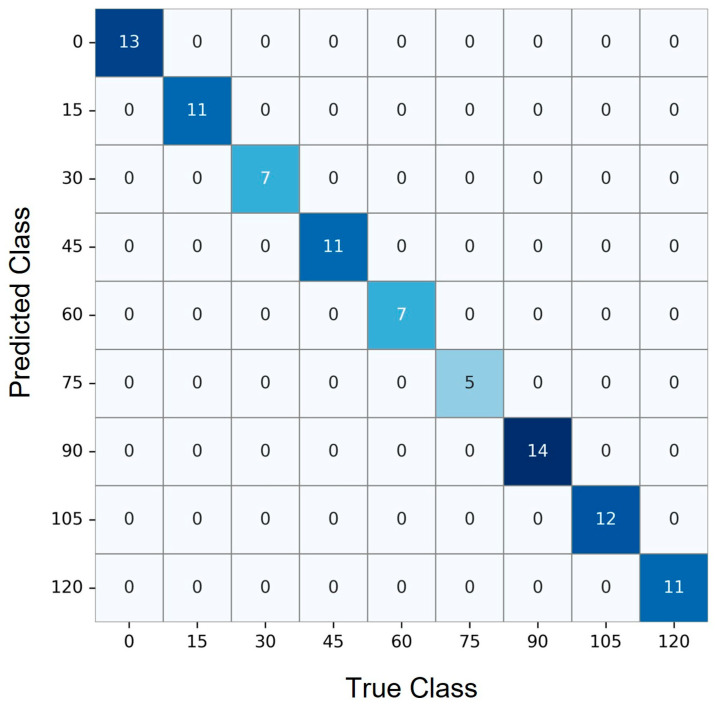
Confusion matrix showing accurate interval classification of time points across nine discrete 15-min steps. Diagonal dominance reflects strong model performance in predicting true elapsed times.

The confusion matrix [40] shown in Figure 4 displays the performance of the time regression model after rounding continuous predictions to the nearest 15-min interval. Each row corresponds to the true elapsed time class, while each column represents the predicted class. A strong diagonal pattern is evident, with the majority of predictions falling on the diagonal cells, indicating high agreement between predicted and actual time intervals. For instance, the model correctly predicted 13 samples in the 0-min class, 11 in the 15-min class, and maintained this trend across later intervals, including 14, 12, and 11 correct predictions in the 90-, 105-, and 120-min classes, respectively.

**Figure 5:**
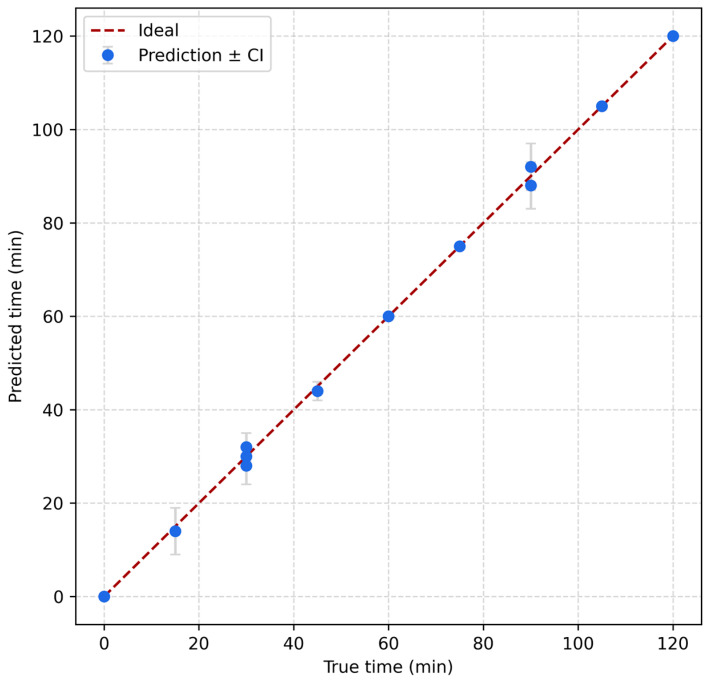
Predicted versus true incubation times using Gaussian Process Regression, with 95% confidence intervals shown as vertical error bars.

To evaluate the model performance, Figure 5 presents the predicted versus true time values for the test set using GPR, with vertical error bars indicating the 95% confidence intervals for each prediction. The data points closely follow the identity line (y = x), suggesting strong agreement between predicted and actual values. The narrow confidence intervals further demonstrate the model’s reliability and low uncertainty, particularly across the full range of incubation times from 0 to 120 min. This alignment confirms the suitability of GPR for accurately estimating yeast growth stages from impedance-derived features. The model exhibited strong regression performance on the test set (*n* = 91), achieving a root mean square error (RMSE) of 0.79 min and a mean absolute error (MAE) of 0.45 min. The coefficient of determination (R^2^ = 0.9996, reporting *p* < 10^−12^) and Pearson correlation (r = 0.9998) indicated excellent agreement with ground truth time labels. Residual analysis showed minimal systematic bias, with a mean error of −0.095 min and a standard deviation of 0.79 min. When continuous outputs were rounded into 15-min intervals, the model maintained 100% classification accuracy across all discrete time bins from 0 to 120 min, confirming its ability to reliably estimate yeast culture progression stages. Bootstrap-based uncertainty quantification yielded 95% confidence intervals for RMSE between [0.48, 1.07] minutes and MAE between [0.34, 0.59] minutes over 500 resampled iterations, reinforcing its predictive robustness under variable sampling. The computational efficiency of the model further supports its deployment potential: training was completed in under two seconds, and inference across all test samples required less than 30 milliseconds in total, with an average latency of 0.30 milliseconds per sample. The model’s compact memory footprint (~1.1 MB) suggests compatibility with embedded systems and real-time bioprocessing environments. While energy consumption per inference is hardware-dependent, it can be approximated by the relation E ≈ P × t, where P is the device power and t is the latency per inference.

## 4. Benchmarking

Several methods exist for estimating yeast cell concentration, each with trade-offs in cost, time, and scalability. Spectrophotometry estimates yeast concentration by measuring turbidity caused by light scattering [41]. It is commonly used for quick monitoring of microbial growth [42]. However, accurate quantification requires calibration against direct cell counts [43]. Hemocytometer counting uses a microscope to directly count cells in a chamber [44]. It gives accurate concentration measurements but is time-consuming and not scalable for high-throughput analysis [45]. Flow cytometry counts yeast cells by detecting light scatter and fluorescence as they pass through a laser beam [46]. It provides rapid, high-throughput measurement of total and viable cell populations [47]. Colony-forming unit (CFU) assays estimate viable cell concentration by plating diluted samples and counting colonies after incubation [48]. Although biologically relevant, the method is time-consuming and low-throughput [49]. Electrical impedance detects yeast cells by sensing resistance changes as they pass through an aperture [50]. It enables real-time, label-free detection and is suitable for automation [51]. However, yeast-specific impedance studies are still relatively limited. Automated image-based counting uses software to analyze microscope images and count cells [52]. Once trained, it enables fast and scalable analysis [53]. This approach has been successfully applied to yeast cell quantification in research settings. Metabolic assays estimate cell concentration by detecting biochemical activity, often through color or fluorescence signals [54]. These methods, such as resazurin-based assays, are common in yeast studies. However, they require calibration and are limited by reagent cost and stability [55]. The various yeast quantification techniques differ significantly in cost, time, effort, power requirements, and equipment setup; these differences are summarized in Table 2. The proposed AI-assisted impedance method addresses a critical gap in current yeast quantification strategies by combining rapid, label-free, and non-optical detection with low system complexity and moderate cost. It offers superior throughput compared to metabolic or plating assays, enhanced scalability over manual counting, and reduced instrumentation demands relative to flow cytometry and image-based systems. This balance of speed, accuracy, and operational efficiency makes it particularly well-suited for real-time monitoring in bioprocessing and industrial fermentation settings.

## 5. Digital Twin Framework for Yeast Cell Monitoring

The proposed AI-enhanced impedance platform establishes a real-time digital twin for yeast culture monitoring [56]. This digital twin acts as a continuously updated virtual counterpart of the physical system, dynamically synchronized with live experimental data [57]. This digital twin integrates the physical biosensor data stream with a cyber layer driven by trained AI models, facilitating real-time predictive monitoring and adaptive control of yeast fermentation. Multi-frequency impedance spectroscopy is employed using a two-electrode configuration. Both magnitude and phase signals are recorded, capturing responses associated with membrane polarization, intracellular conductivity, and overall biomass. These electrical parameters exhibit sensitivity to physiological changes during cell growth [58], making them highly suitable for label-free, non-invasive monitoring. The raw impedance signals undergo statistical smoothing and normalization. Feature engineering techniques are applied to extract robust descriptors, including combined magnitude–phase metrics (e.g., |Z|*θ*), which improve the biological interpretability of the electrical data. These features are then fed into trained machine learning models. Ensemble classification is used to identify discrete growth phases, while Gaussian Process Regression (GPR) estimates biomass or optical density continuously. GPR further provides confidence intervals for each prediction, enabling uncertainty-aware inference that enhances reliability in bioprocess decision-making.

**Figure 6:**
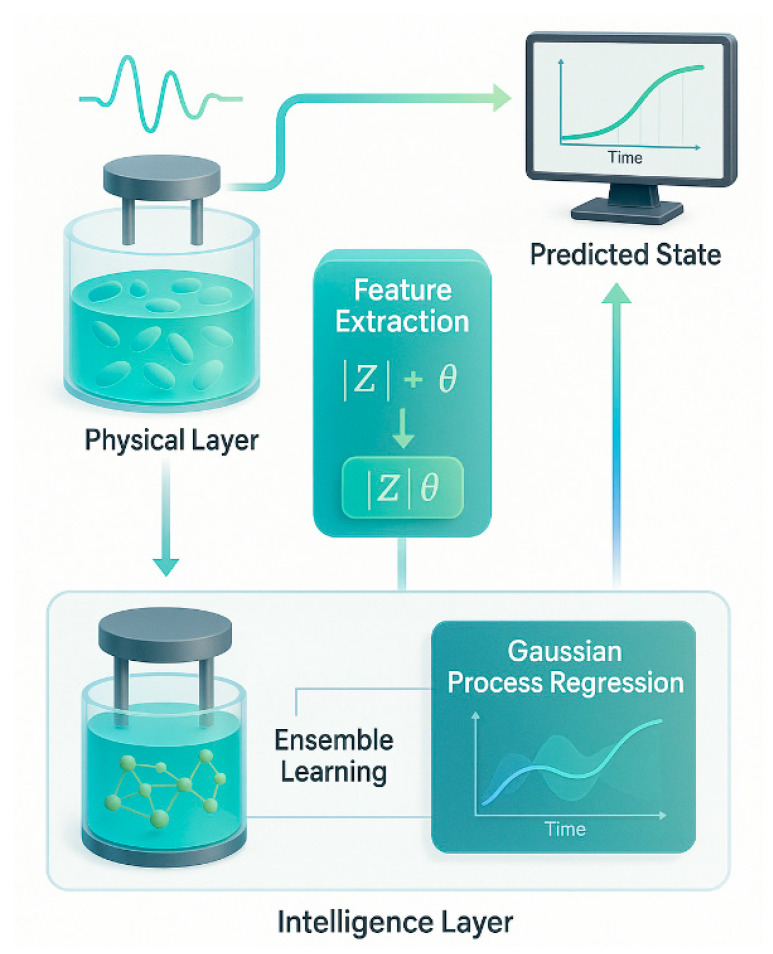
Proposed AI-driven digital twin system for real-time yeast monitoring.

The digital twin architecture forms a closed-loop system as shown in Figure 6, that maintains real-time alignment between the physical yeast culture and its virtual model [59]. This architecture supports predictive monitoring, trend forecasting, and early anomaly detection [60]. It also accommodates variability in growth conditions, including differences in yeast strains, initial cell concentrations, and medium composition [61]. Unlike conventional optical methods, the proposed impedance-based platform operates without reliance on labeling or turbidity-based optics. It functions effectively across a broad range of environmental and biological scenarios [62].

Furthermore, the system’s low-cost and compact hardware makes it adaptable for both laboratory-scale experiments and industrial-scale fermenters. Its versatility enables integration into scalable, automated bioprocessing pipelines. As illustrated in Figure 6, the system begins at the physical layer, where yeast cultures are probed using multi-frequency impedance spectroscopy. The two-electrode system captures both |Z| (magnitude) and θ (phase). This data is passed to the feature extraction module, which computes engineered metrics such as |Z|θ. These features enter the intelligence layer, where ensemble models classify discrete growth states and GPR maps the time evolution of biomass. The predicted state—biologically meaningful parameters such as OD_600_—is rendered on a digital interface, completing the real-time synchronization between physical and virtual domains.

## 6. Conclusions

This study presents a novel AI-integrated electrochemical impedance platform for accurate, label-free monitoring of yeast cell growth. By leveraging multi-frequency impedance data and engineered signal features—including a magnitude–phase interaction metric—we captured the underlying bioelectrical signatures of cellular proliferation. Gaussian Process Regression enabled continuous prediction of yeast growth with exceptional accuracy (R^2^ = 0.9996; RMSE = 0.79 min), while discretized outputs achieved perfect classification across time intervals, highlighting both regression fidelity and stage-wise interpretability.

The use of artificial intelligence was not merely additive, but essential. Traditional methods—whether optical or statistical—are constrained by variability in initial conditions and the nonlinearity of biological systems. In contrast, our AI model learned invariant mappings between impedance features and cell concentration, robustly generalizing across experimental runs with varying starting densities. This ability to extract latent biological trends from noisy, high-dimensional electrochemical data positions AI as a critical enabler for real-time bioprocess monitoring.

Beyond performance, the system’s low latency, minimal computational load, and optical independence make it ideally suited for embedded, scalable deployment in fermentation and industrial biotechnology. The integration of machine learning with electrical biosensing lays the foundation for real-time digital twins—self-updating virtual replicas of living systems—that offer predictive, adaptive, and automated control in dynamic environments.

Together, these contributions demonstrate a significant step toward next-generation intelligent biosensors capable of redefining microbial monitoring in both research and industry.

## Figures and Tables

**Table 1:** Random subset of ten impedance spectra entries sampled from the statistical dataset used for machine learning. Each row corresponds to a unique excitation frequency (1.26 Hz to 6.3 kHz), with columns reporting mean and standard deviation of both magnitude and phase across selected time points (t_0_…t_8_). These engineered features formed the core input space for regression and classification models, capturing electrical changes associated with yeast proliferation.

Freq (Hz)	5.008	9.931	2.504	6328	398	1.259	63.075	100	794	1995
t0_Mean_Mag	10,850.33	7081.33	16,222	138.46	548	19,465.67	937.2	670.7	289.9	204.3
t0_SD_Mag	7361.56	5122.36	9974.58	82.27	449.84	11,475.38	689.15	491.79	205.39	126.94
t0_Mean_Phase	−55.54	−58.68	−51.64	−23.53	−56.57	−48.99	−42.58	−46.46	−54.15	−50.61
t0_SD_Phase	9.85	9.76	9.46	12.82	2.17	7.77	3.27	2.65	1.45	2.43
…	…	…	…	…	…	…	…	…	…	…
t8_Mean_Mag	8322.33	5108	13,118	100.26	296.5	15,238.33	534.8	384.5	144	112.43
t8_SD_Mag	4541.56	3039.25	6403.56	31.67	204.34	8391.09	387.65	281.66	98.56	65.88
t8_Mean_Phase	−62.62	−66.44	−57.49	−15.3	−53.89	−55.66	−50.53	−52.1	−51.59	−46.55
t8_SD_Phase	8.13	6.99	8.91	10.69	7.68	7.94	6.75	5.56	4.98	5.49

**Table 2:** Summary of yeast cell concentration techniques.

Method	Cost	Time	Effort	Power	Setup	Speed
Spectrophotometry	L	Seconds	L	L	Simple	F
Hemocytometer	VL	10–20 min/sample	H	None	Basic (microscope)	S
Flow Cytometry	VH	5–10 min/sample	L	H	Complex lab setup	F
Dry Weight	L	Hours	H	M	Moderate (oven)	S
CFU (Plating)	M	24–48 h	VH	M	Basic lab	VS
Electrical Impedance	M	Seconds–minutes	L	M	Needs circuit design	F
AI Image Analysis	V	Seconds	L	M–H	Imaging + ML required	F
Metabolic Assays	M	30–60 min	M	L	Reader and reagents	M

VL: Very Low; L: Low; M: Medium/Moderate; H: High; VH: Very High; V: Variable; VS: Very Slow; S: Slow; F: Fast.

## Data Availability

Dataset available on request from the authors.

## Abbreviations

The following abbreviations are used in this manuscript:

OD	Optical density
EIA	Electrical impedance analysis
YPD	Yeast extract Peptone Dextrose
GPR	Gaussian Process Regression
RMSE	Root Mean Square Error
